# Supplementary material for: Mini-PCDH15 gene therapy rescues hearing in a mouse model of Usher syndrome type 1F
Source: Nat Commun. 2023 Apr 26;14:2400. doi: 10.1038/s41467-023-38038-y (PMC10133396; doi:10.1038/s41467-023-38038-y)
Supplement: Supplementary file 1 — Supplementary Information [file 41467_2023_38038_MOESM1_ESM.pdf]

Supplementary Information for

Mini-PCDH15 gene therapy rescues hearing in a mouse model of Usher syndrome type 1F

Maryna V Ivanchenko[1], Daniel M Hathaway[1,2], Alex J Klein[1], Bifeng Pan[1], Olga Strelkova[2], Pedro De-la-Torre[2], Xudong Wu[1], Cole W Peters[1], Eric M Mulhall[1], Kevin T Booth[1], Corey Goldstein[1], Joseph Brower[2], Marcos Sotomayor[3], Artur A Indzhykulian[2], David P Corey[1]

1. Department of Neurobiology, Harvard Medical School, Boston, MA, USA

2. Department of Otolaryngology - Head and Neck Surgery, Harvard Medical School and Massachusetts Eye and Ear, Boston, MA, USA

3. Department of Chemistry and Biochemistry, The Ohio State University, Columbus, OH, USA

Corresponding author

David P Corey (email: david_corey@hms.harvard.edu)

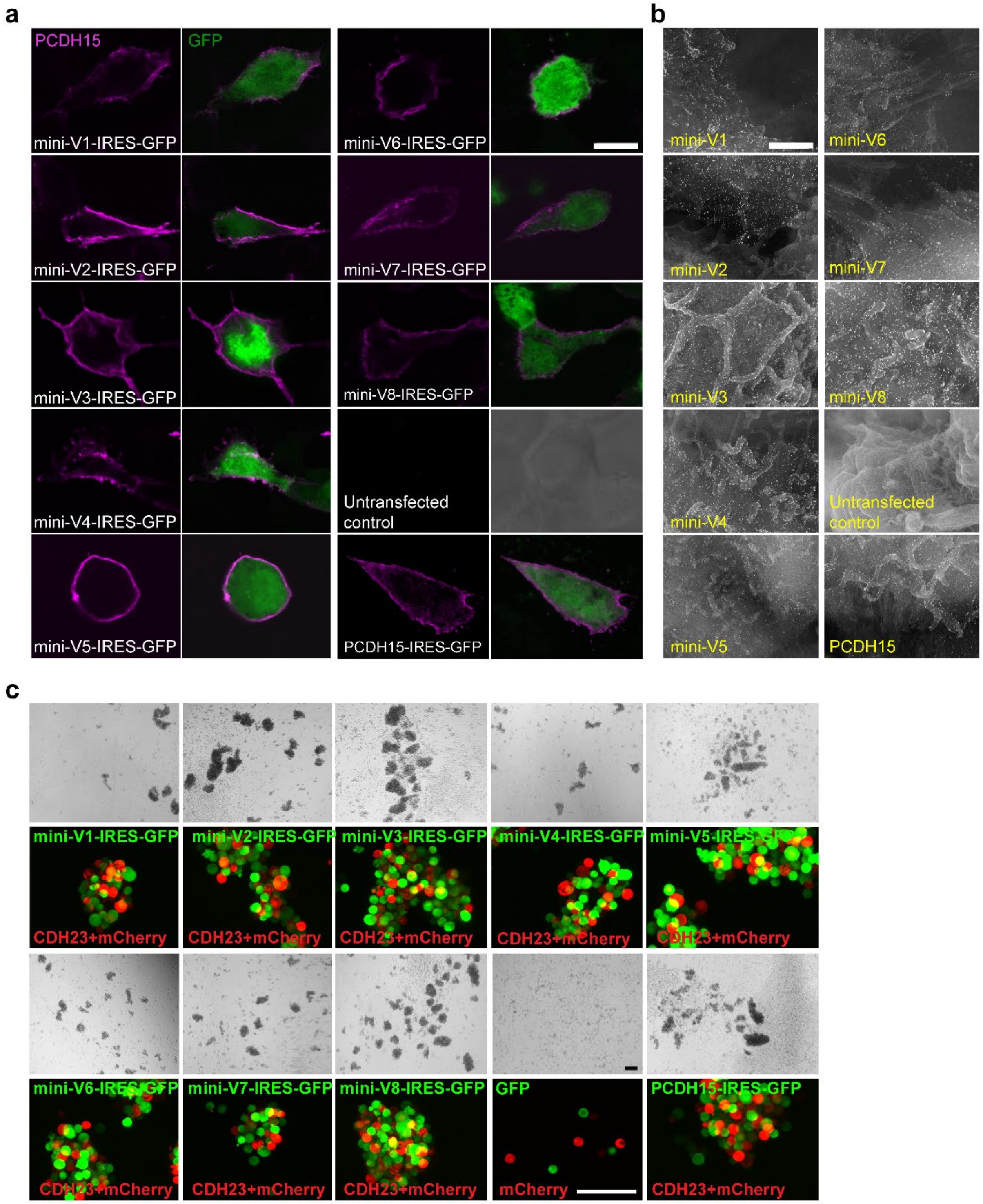

Supplementary Fig. 1.

Proper targeting and binding of miniPCDH15s. Mini-PCDH15-IRES-eGFP and full length-PCDH15-IRES-eGFP plasmids were transfected into N-cadherin knockout HEK293 cells. **a** Immunostaining was performed with anti-PCDH15 antibodies (magenta). Representative confocal images demonstrate normal cell membrane trafficking of mini-PCDH15s similar to full-length-PCDH15. **b** Representative immunogold scanning electron micrographs show extracellular labeling of mini-PCDH15s, indicating normal protein transport to the cell membrane. **c** Binding of mini-PCDH15s to CDH23 using cell aggregation assay. Cells expressing full-length PCDH15 or mini-PCDH15 (green) aggregate with cells expressing CDH23 (red) when mixed together on a rotating shaker. Scale bars, **a** 20 μm, **b** 1 μm, **c** 50 μm.

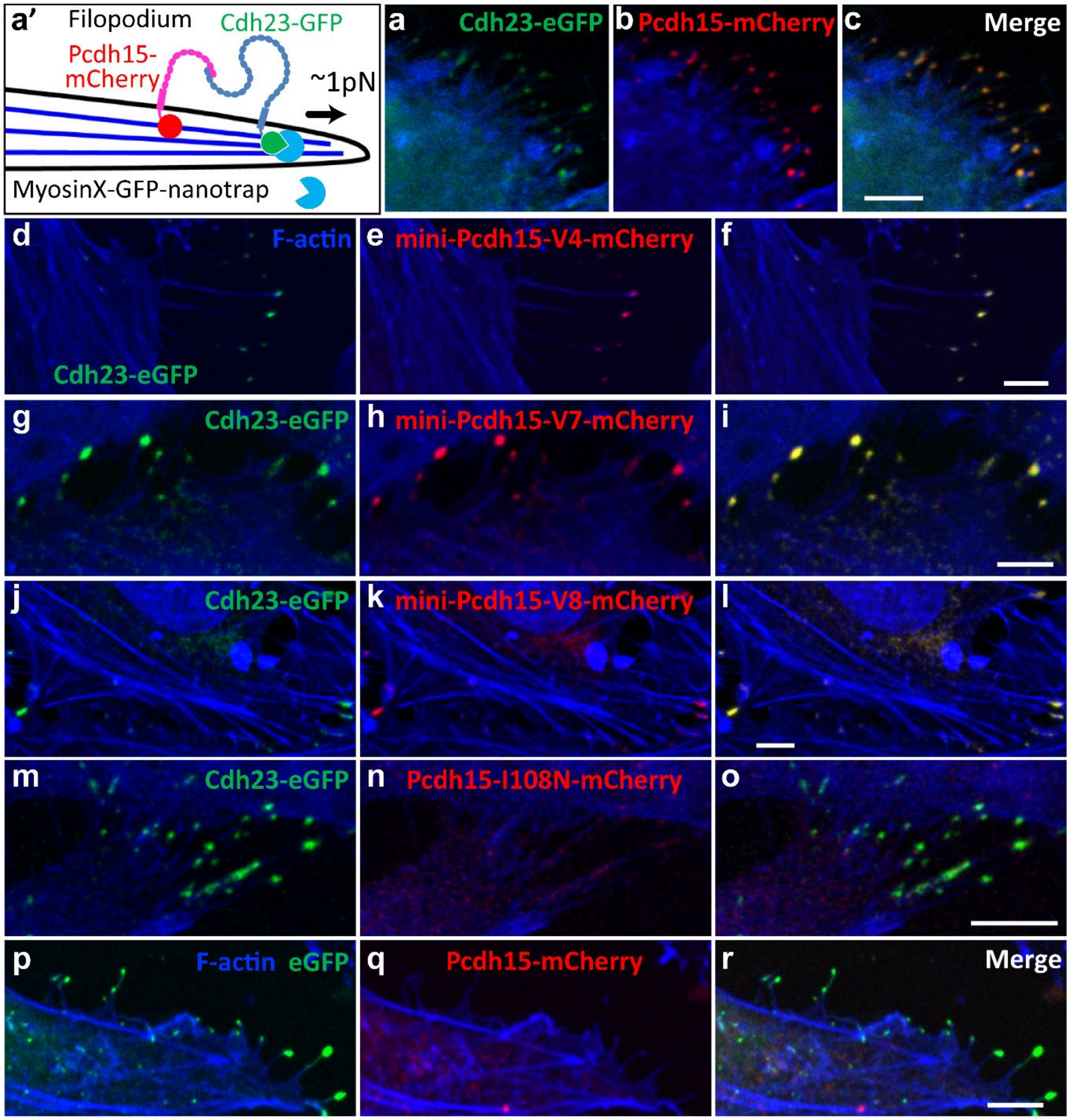

Supplementary Fig. 2.

NanoSPD assay to evaluate PCDH15 interaction with CDH23 under force. a Schematic of myosin motor and linked cadherins. MYO10-GFP$_{nanotrap}$, CDH23-GFP (bait) and PCDH15-mCherry (prey) are co-transfected in HeLa cells. MYO10-GFP$_{nanotrap}$ binds to eGFP and pulls CDH23-GFP along the actin core to the filopodia. When PCDH15-mCherry is bound to CDH23-eGFP, it is also transported to the tips. **a**-**c** Positive controls (n=6 independent experiments). **a** MYO10 pulled CDH23-eGFP to the tips of filopodia. **b** PCDH15-mCherry was drawn to the tips by CDH23-eGFP bound to MYO10-GFP$_{nanotrap}$. **c**, Merged image shows colocalization. **d**-**r**, Experimental. **d**-**f** Mini-PCDH15-V4 (n=5 independent experiments), **g**-**i** mini-PCDH15-V7 (n=3 independent experiments) and **j**-**l** mini-PCDH15-V8 (n=4 independent experiments) were drawn to the tips by CDH23-eGFP bound to MYO10-GFP$_{nanotrap}$. **m**-**r** Negative controls. **m**-**o** PCDH15-I108N was not drawn to the tips by CDH23-eGFP (n=3 independent experiments). **p**-**r** eGFP alone was unable to draw PCDH15-mCherry to the tips of filopodia suggesting the interaction is mediated by the presence of CDH23 (n=5 independent experiments). Scale bars, 5 μm.

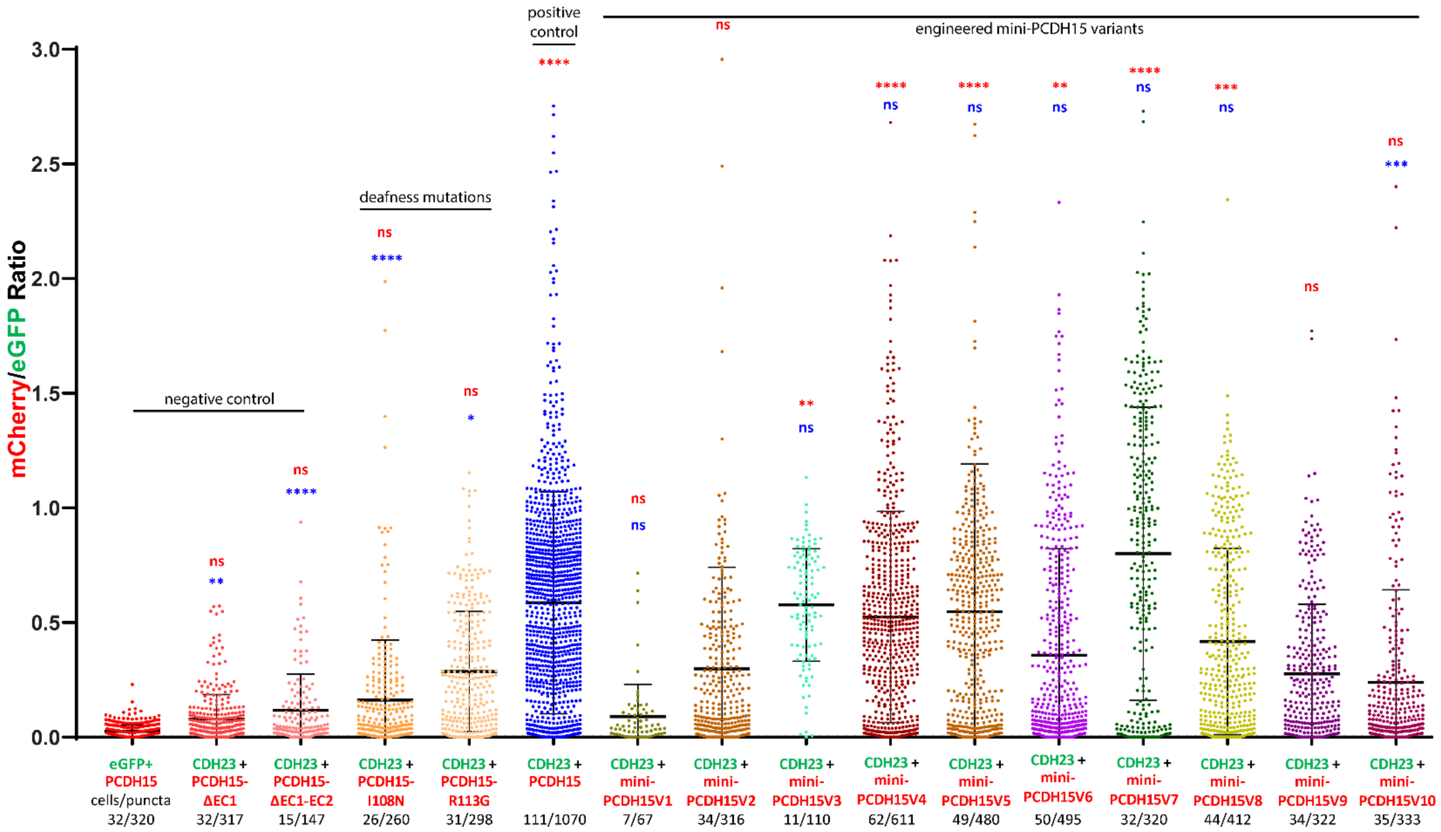

Supplementary Fig. 3.

Quantification of the NanoSPD assay to evaluate PCDH15 interaction with CDH23 under force. Triple-transfected HeLa cells, expressing MYO10-GFP-nanotrap, eGFP or CDH23-eGFP along with a PCDH15 variant, were imaged and their fluorescent puncta quantified. Up to 10 puncta per cell were used to measure the mCherry/eGFP fluorescence ratio. The number of cells/puncta used in this analysis is summarized for each combination of constructs: eGFP+PCDH15-mCherry (n=32/320), CDH23-eGFP+PCDH15-mCherry (n=111/1070), CDH23-eGFP+PCDH15ΔEC1-mCherry (n=32/317), CDH23-eGFP+PCDH15ΔEC1-EC2-mCherry (n=15/147), CDH23-eGFP+PCDH15-R113G-mCherry (n=31/298), CDH23-eGFP+PCDH15-I108N-mCherry (n=26/260), CDH23-eGFP+mini-PCDH15-V1 (n=7/67), CDH23-eGFP+mini-PCDH15-V2 (n=34/316), CDH23-eGFP+mini-PCDH15-V3 (n=11/110), CDH23-eGFP+mini-PCDH15-V4 (n=62/611), CDH23-eGFP+mini-PCDH15-V5 (n=49/408), CDH23-eGFP+mini-PCDH15-V6 (n=50/495), CDH23-eGFP+mini-PCDH15-V7 (n=32/320), CDH23-eGFP+mini-PCDH15-V8 (n=44/412), CDH23-eGFP+mini-PCDH15-V9 (n=34/322), CDH23-eGFP+mini-PCDH15-V10 (n=35/333). Low ratios were seen with negative control combinations: eGFP+PCDH15, CDH23-eGFP + PCDH15ΔEC1-EC2 (lacking the EC1 and EC2 repeats critical for interaction with CDH23), CDH23-eGFP + PCDH15ΔEC1 (lacking EC1), and PCDH15 with known deafness mutations (I108N and R113G). High ratios were seen with the positive control: CDH23-eGFP + PCDH15 (full length). Ten engineered mini-PCDH15 variants performed at different levels. Mini-PCDH15 versions 3, 4, 5, 6, 7, and 8 were transported as well as the positive control. As additional negative controls, we tested two extremely short mini-PCDH15 variants: V9 and V10. Mini-PCDH15-V9 (EC1-EC2-EC3---EC11-MAD12) lacks seven EC repeats, while mini-PCDH15-V10 (EC1-EC2---EC11-MAD12) lacks eight; both lack the EC3 domain required for parallel dimerization of PCDH15. Neither performed well in the Nano-SPD assay, showing that EC3 and EC10 are also needed for function. All cDNA constructs were full sequence verified before and at the end of experiments. Data are shown as mean ± SD. The statistical significance was evaluated using multiple comparisons nested one-way ANOVA, and corrected for multiple comparisons using Tukey test. Significance is shown with "ns" or asterisks (ns – not significant, * - $p<0.05$, ** - $p<0.01$, *** - $p<0.001$, **** - $p<0.0001$) with red symbols relative to the negative control lacking CDH23 and blue symbols relative to the positive control with both CDH23 and PCDH15.

P values:

eGFP+PCDH15-mCherry vs. CDH23-eGFP+PCDH15-mCherry	$p<0.0001$ (****)
eGFP+PCDH15-mCherry vs. CDH23-eGFP+PCDH15-I108N	$p>0.99$ (ns)
eGFP+PCDH15-mCherry vs. CDH23-eGFP+PCDH15-R113G	$p=0.24$ (ns)
eGFP+PCDH15-mCherry vs. CDH23-eGFP+PCDH15ΔEC1	$p>0.99$ (ns)
eGFP+PCDH15-mCherry vs. CDH23-eGFP+PCDH15ΔEC1-EC2	$p>0.99$ (ns)
eGFP+PCDH15-mCherry vs. CDH23-eGFP+mini-PCDH15-V2	$p=0.16$ (ns)
eGFP+PCDH15-mCherry vs. CDH23-eGFP+mini-PCDH15-V3,	$p=0.003$ (**)
eGFP+PCDH15-mCherry vs. CDH23-eGFP+mini-PCDH15-V4	$p<0.0001$(****)
eGFP+PCDH15-mCherry vs. CDH23-eGFP+mini-PCDH15-V5	$p<0.0001$(****)
eGFP+PCDH15-mCherry vs. CDH23-eGFP+mini-PCDH15-V6	$p=0.004$ (**)
eGFP+PCDH15-mCherry vs. CDH23-eGFP+mini-PCDH15-V7	$p<0.0001$(****)
eGFP+PCDH15-mCherry vs. CDH23-eGFP+mini-PCDH15-V8	$p=0.0005$ (***)
eGFP+PCDH15-mCherry vs. CDH23-eGFP+mini-PCDH15-V9	$p=0.31$ (ns)
eGFP+PCDH15-mCherry vs. CDH23-eGFP+mini-PCDH15-V10	$p=0.60$ (ns)
eGFP+PCDH15-mCherry vs. CDH23-eGFP+mini-PCDH15-V1	$p>0.99$ (ns)

CDH23-eGFP +PCDH15-mCherry vs. CDH23-eGFP+PCDH15-I108N	$p<0.0001$(****)
CDH23-eGFP +PCDH15-mCherry vs. CDH23-eGFP+PCDH15-R113G	$p=0.016$ (*)
CDH23-eGFP +PCDH15-mCherry vs. CDH23-eGFP+PCDH15ΔEC1	$p<0.0001$(****)
CDH23-eGFP+PCDH15-mCherry vs. CDH23-eGFP+PCDH15ΔEC1-EC2	$p=0.001$ (**)
CDH23-eGFP +PCDH15-mCherry vs. CDH23-eGFP+mini-PCDH15-V2	$p=0.015$ (*)
CDH23-eGFP +PCDH15-mCherry vs. CDH23-eGFP+mini-PCDH15-V3,	$p>0.99$ (ns),
CDH23-eGFP +PCDH15-mCherry vs. CDH23-eGFP+mini-PCDH15-V4	$p>0.99$ (ns)
CDH23-eGFP +PCDH15-mCherry vs. CDH23-eGFP+mini-PCDH15-V5	$p>0.99$ (ns)
CDH23-eGFP +PCDH15-mCherry vs. CDH23-eGFP+mini-PCDH15-V6	$p=0.064$ (ns)
CDH23-eGFP +PCDH15-mCherry vs. CDH23-eGFP+mini-PCDH15-V7	$p=0.25$ (ns)
CDH23-eGFP +PCDH15-mCherry vs. CDH23-eGFP+mini-PCDH15-V8	$p=0.5$ (ns)
CDH23-eGFP +PCDH15-mCherry vs. CDH23-eGFP+mini-PCDH15-V9	$p=0.004$ (**)
CDH23-eGFP +PCDH15-mCherry vs. CDH23-eGFP+mini-PCDH15-V10	$p=0.0004$ (***)
CDH23-eGFP +PCDH15-mCherry vs. CDH23-eGFP+mini-PCDH15-V1	$p=0.092$ (ns).

Source data are provided as a Source Data file

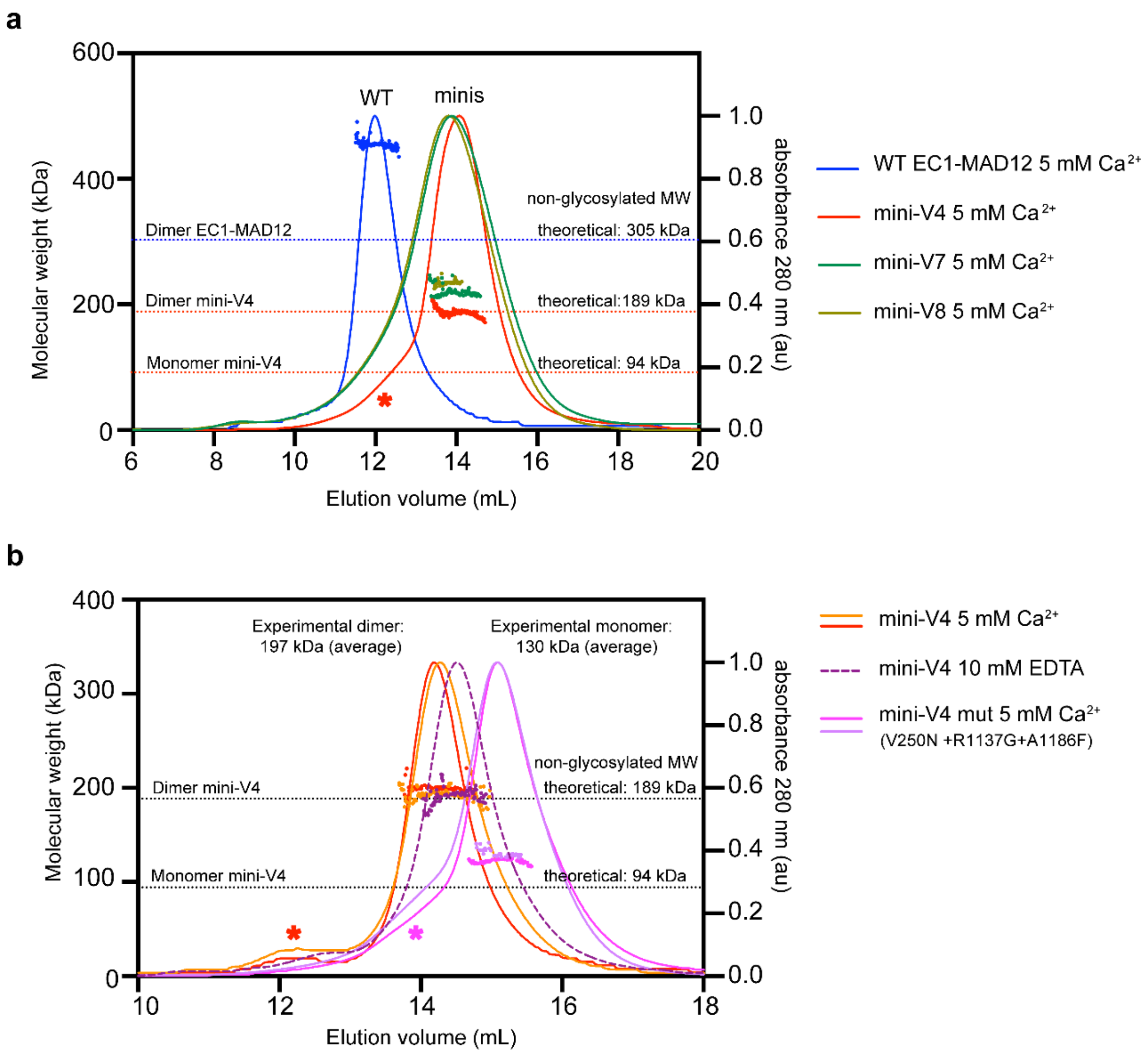

Supplementary Fig. 4.

Dimerization in solution of glycosylated PCDH15 extracellular domains of WT EC1-MAD12 and mini-PCDH15. a SEC-MALS analysis of mouse PCDH15 EC1-MAD12 ex12a- extracellular domain (blue curve), expressed *in vitro* and purified in the presence of 5 mM Ca^{2+} using a Superose-6 10/300 Increase column. Theoretical monomeric and dimeric molecular weights (MW) for non-glycosylated WT PCDH15 EC1-MAD12 are 153 kDa and 305 kDa, respectively (dimer: blue dashed line). The WT EC1-MAD12 protein (blue) showed a monodisperse peak eluting at 11.9 mL with MW = 474 kDa, suggesting a glycosylated dimer. Higher molecular weight oligomers are also possible. Mini-PCDH15 extracellular domains had a lower molecular weight than WT, as expected, but the molecular weights also indicated dimerization: mini-PCDH15-V4 (red) eluted at 14.0 mL with MW = 200 kDa; mini-PCDH15-V7 (green) at 14.0 mL with MW=205 kDa; and mini-PCDH15-V8 (olive) at 13.9 mL with MW = 241 kDa. Higher MW forms were present (shoulder signal ~12 mL, red asterisk). Solid lines represent UV absorbance at 280 nm in absorbance units (au) (right-hand axis). Scattering profiles are represented by horizontal dots (left axis); horizontal dashed lines (in red) indicate theoretical MW for non-glycosylated monomers (94 kDa) and dimers (189 kDa) of mini-PCDH15-V4 as a reference. Overall, SEC-MALS indicated that all proteins tested are dimeric in solution with possible higher molecular weight oligomers. **b** SEC-MALS of mouse mini-PCDH15-V4 showed one main peak at 14.2 mL when protein was purified in the presence of 5 mM Ca^{2+} (red and orange curves; two replicates obtained from independently transfected cell cultures; MW = 193 kDa and MW =200 kDa). Combined replicate samples were mixed and reanalyzed following Ca^{2+} chelation by adding

10 mM EDTA to the SEC buffer (dashed purple curve; elution volume at 14.6 mL and MW = 192 kDa). The scattering profiles and slightly shifted elution volume peak at 14.6 mL indicated that the mini-PCDH15-V4 (MW = 200 kDa) was still dimeric in the absence of Ca^{2+}. In contrast, an engineered mini-PCDH15-V4 triple mutant that disrupts both the EC2-3 and EC11-MAD12 dimerization domains (V250N + Rll37G + A1186F; purified in 5 mM Ca^{2+}) showed elution peaks shifted to 15.1 mL and scattering consistent with a monomer (purple and pink curves, two biological replicates; MW = 129 kDa and 130 kDa, respectively). Average experimental molecular weight values are shown in bold at the top of each peak in panel b. In addition, higher molecular weight forms were present for mini-PCDH15-V4 (shoulder peak at 12-13 mL of elution, red asterisk), and likely for the mutant PCDH15-V4 (shoulder peak ~14 mL, pink asterisk). These results suggest that mini-PCDH15-V4 mimics the parallel dimerization of the native PCDH15 EC1-MAD12 by retaining the EC1-3 and EC11-MAD12 dimeric structure in solution. Source data are provided as a Source Data file.

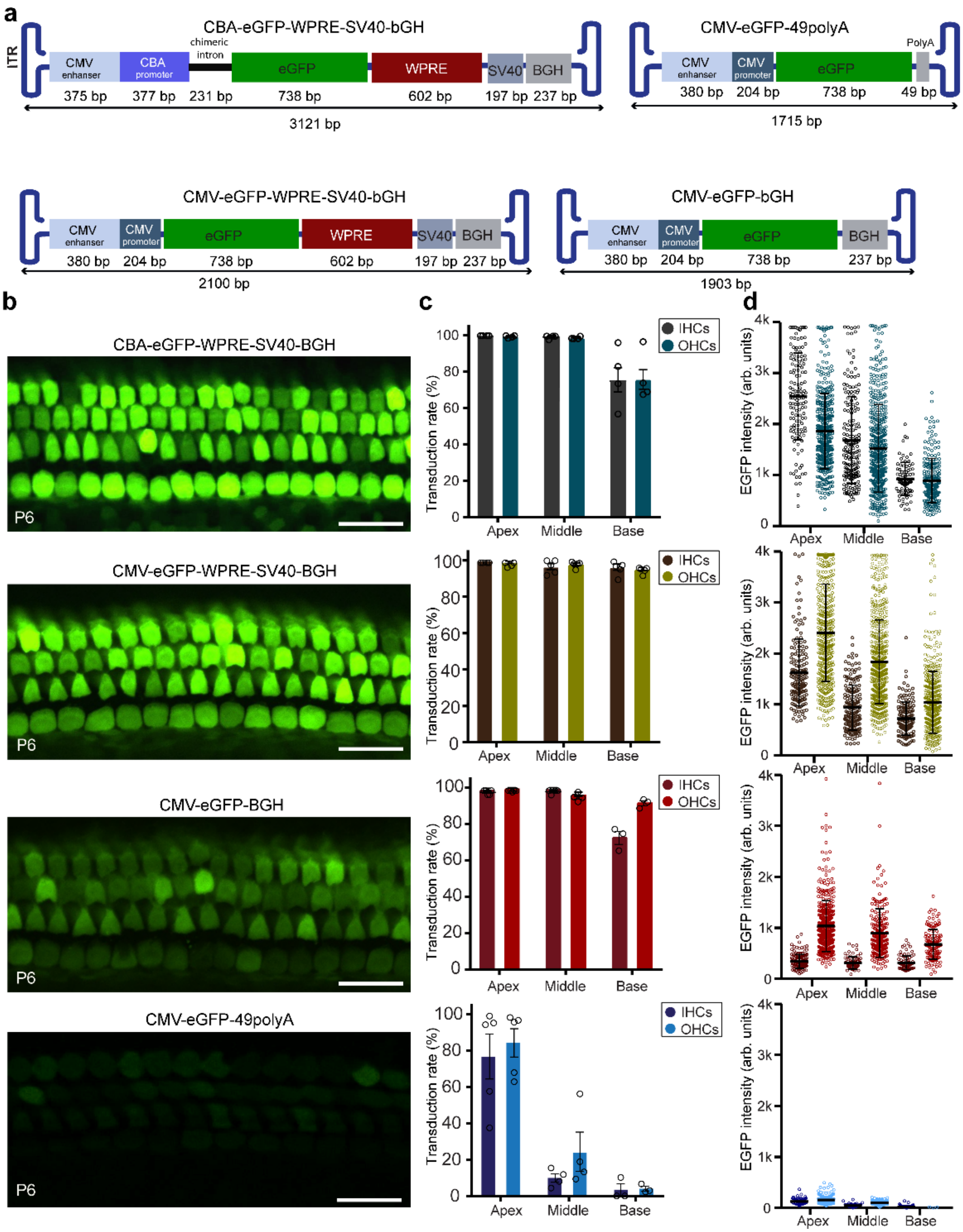

Supplementary Fig. 5.

Optimization of AAV expression cassettes to improve packaging capacity and transgene expression in the cochlea. a Experimental strategy for modifying expression cassettes. Four different constructs were packaged into AAV9-PHP.B and injected ($1x10^{11}$ VG) via the round window membrane (RWM) into neonatal P1 C57BL/6J mouse cochleas. Two different promoters and three different polyadenylation sequences were tested along with a woodchuck hepatitis virus post-transcriptional regulatory element (WPRE) sequence. The length of each DNA sequence is shown below the element. **b** Representative confocal images showing eGFP expression in the organ of Corti (middle turn) of P6 mice injected with one of the four constructs. Scale bar, 20 µm. **c** Transduction efficiency as a proportion of inner hair cells (IHCs) and outer hair cells (OHCs) of the apical, middle, and basal

cochlea turns at P6, from C57BL/6J mice injected with AAV-CBA-eGFP-WPRE-SV40-BGH (n=4), AAV-CMV-eGFP-WPRE-SV40-BGH (n=5), AAV-CBA-eGFP-BGH (n=5), or AAV-CBA-eGFP-49polyA (n=5). Data are presented as mean ± SEM. **d** Quantification of eGFP fluorescence intensity in individual cells in arbitrary units. Cells measured were from the apical, middle, and basal cochlea turns at P6 in C57BL/6J mice injected with AAV-CBA-eGFP-WPRE-SV40-BGH (n=4), AAV-CMV-eGFP-WPRE-SV40-BGH (n=5), AAV-CBA-eGFP-BGH (n=5), or AAV-CBA-eGFP-49polyA (n=5). Data are presented as mean ± SD. Source data are provided as a Source Data file.

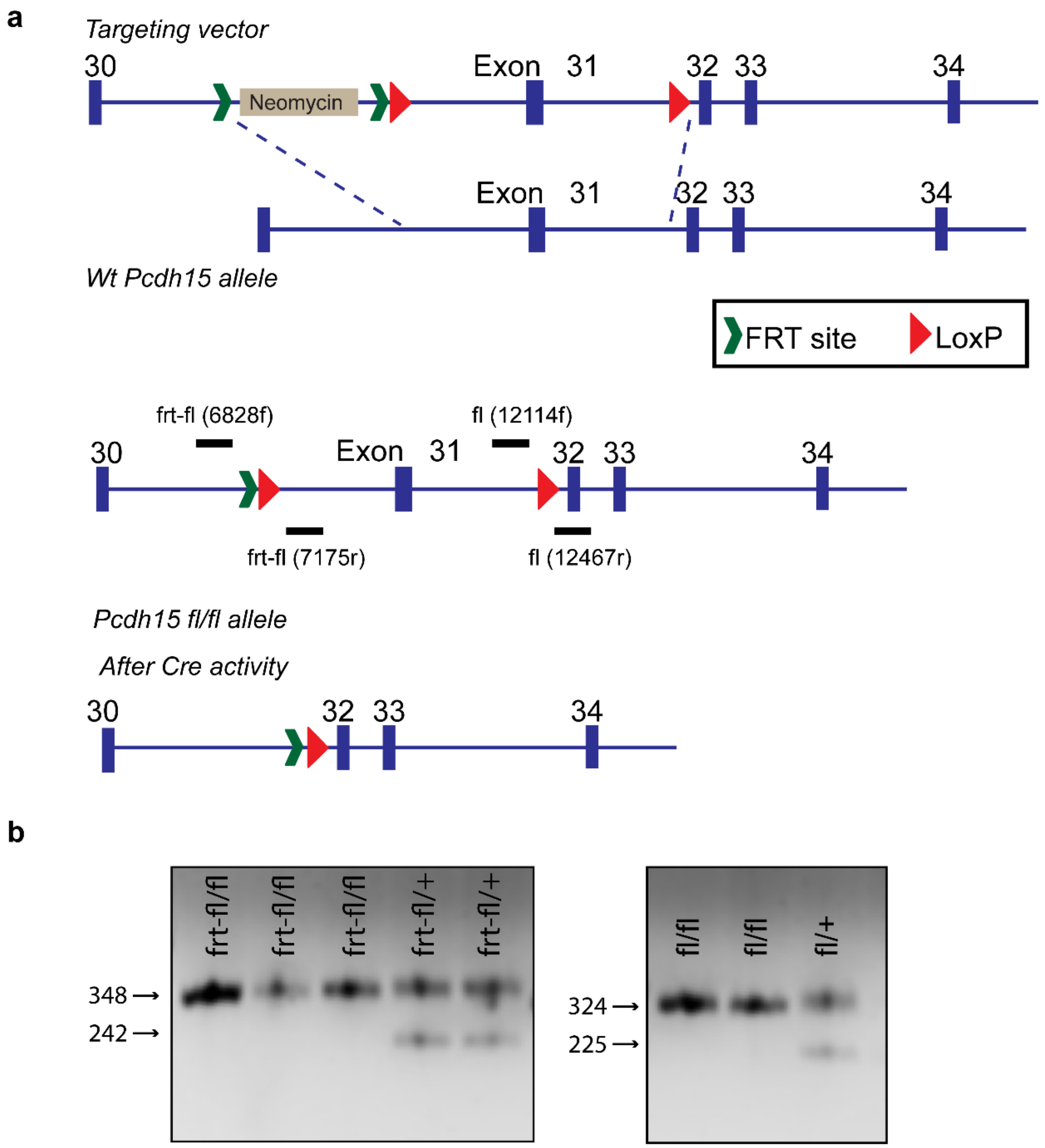

Supplementary Fig. 6.

Generation of conditional knockout mouse model. a Schematic of the recombinant *Pcdh15* locus in conditional knockout mice. A targeting vector was designed in which loxP sites were introduced upstream and downstream from *Pcdh15* exon 31, which encodes the single transmembrane domain. After germline transmission, mice were crossed with 129/Sv mice producing Flp recombinase to remove the FRT-flanked neo cassette. The *Pcdh15*$^{fl/fl}$ mice lack the neo cassette and behave like wild-type (*Pcdh15*$^{+/+}$) mice. **b** The genotyping primers for the *Pcdh15*$^{fl/fl}$ floxed allele were designed to detect the loxP sites using two primer pairs: [cPCDH15-12114f: 5′-ggtttctctgtttctttaac-3′ and cPCDH15-12467r: 5′-gggtttcagttctaataaga-3′] and [cPCDH15-FRT-6828f: 5′-cctcctttatgttccatata-3′ and cPCDH15-FRT-7175r: 5′-ccagaaaattctaaatgcct-3′]. As expected, the floxed allele downstream of exon 31 produced a 324-bp PCR band, whereas the wild-type allele generated a ~225-bp PCR band using primers cPCDH15-12114f and cPCDH15-12467r (right panel). The Frt-floxed allele upstream of exon 31 produced a 348-bp PCR band, whereas the wild-type allele generated a ~242-bp PCR band using primers cPCDH15-FRT-6828f and cPCDH15-FRT-7175r (left panel). All studies were performed on a mixed C57BL/6–129/Sv genetic background. Source data are provided as a Source Data file.

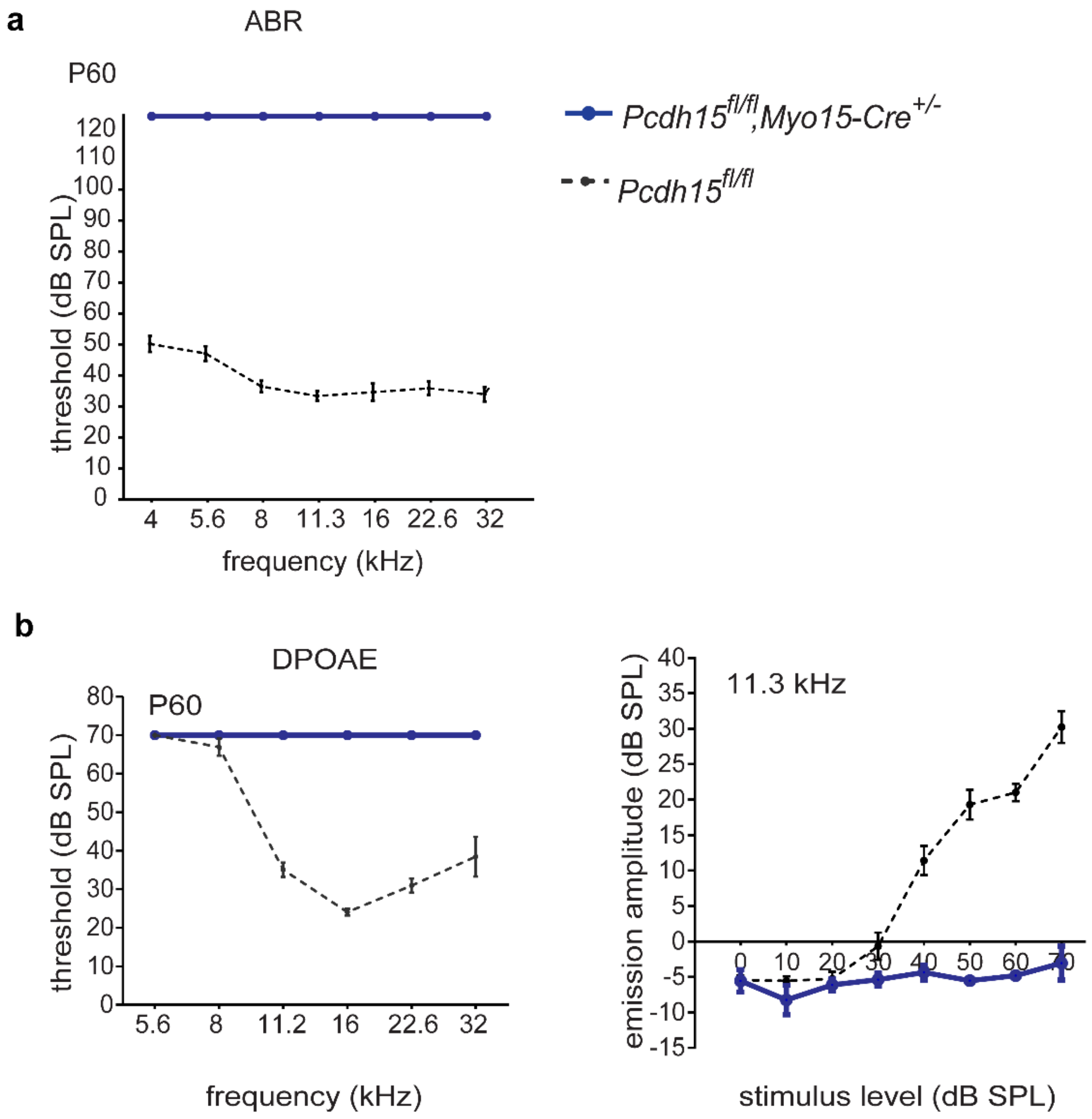

Supplementary Fig. 7.

***Myo15-Cre* conditional knockout mice are profoundly deaf by P60.**

a, b At P60, no auditory brainstem response (ABR) or distortion-product otoacoustic emission (DPOAE) thresholds could be detected in *Pcdh15*$^{fl/fl}$,*Myo15-Cre*$^{+/-}$ mice (n=5), compared to *Pcdh15*$^{fl/fl}$ mice (n=8). Data are presented as mean ± SEM. Source data are provided as a Source Data file.

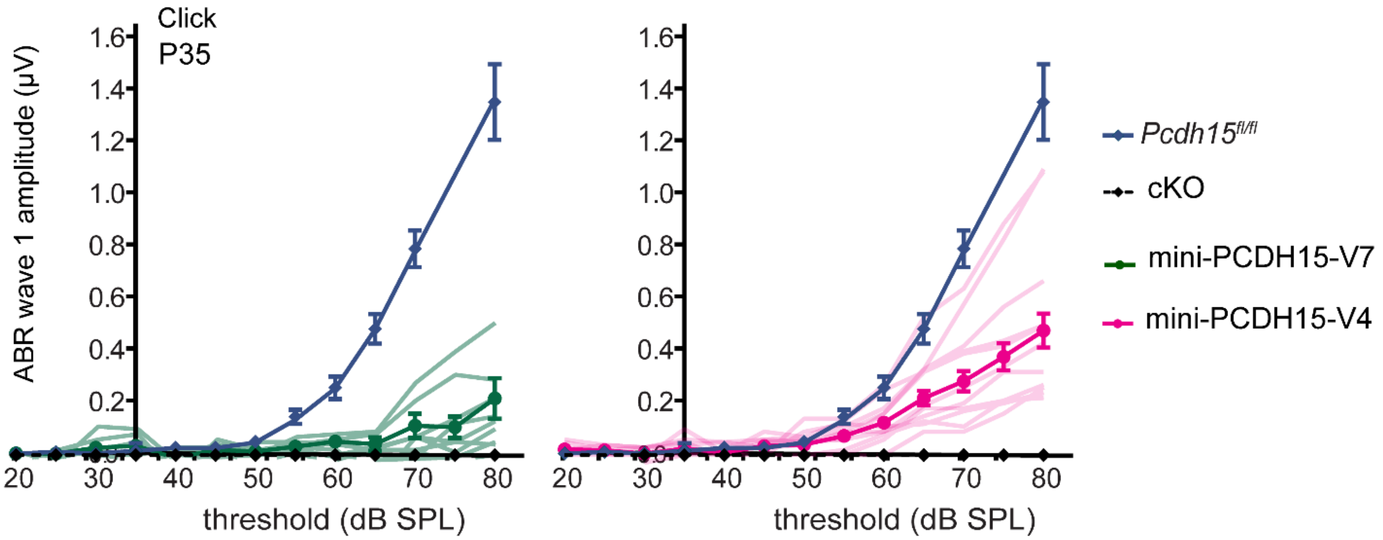

Supplementary Fig. 8.
Average P1 click auditory brainstem response (ABR) amplitudes for P35 uninjected *Pcdh15*$^{fl/fl}$ mice (blue; n=4), for uninjected *Pcdh15*$^{fl/fl}$,*Myo15-Cre*$^{+/-}$(cKO; black; n=10), and for conditional knockout mice injected with AAV-mini-Pcdh15-V4 (pink; n=17) or -V7 (green; n=10). Light traces are amplitudes for individual animals. Averaged data are presented as mean ± SEM. Source data are provided as a Source Data file.

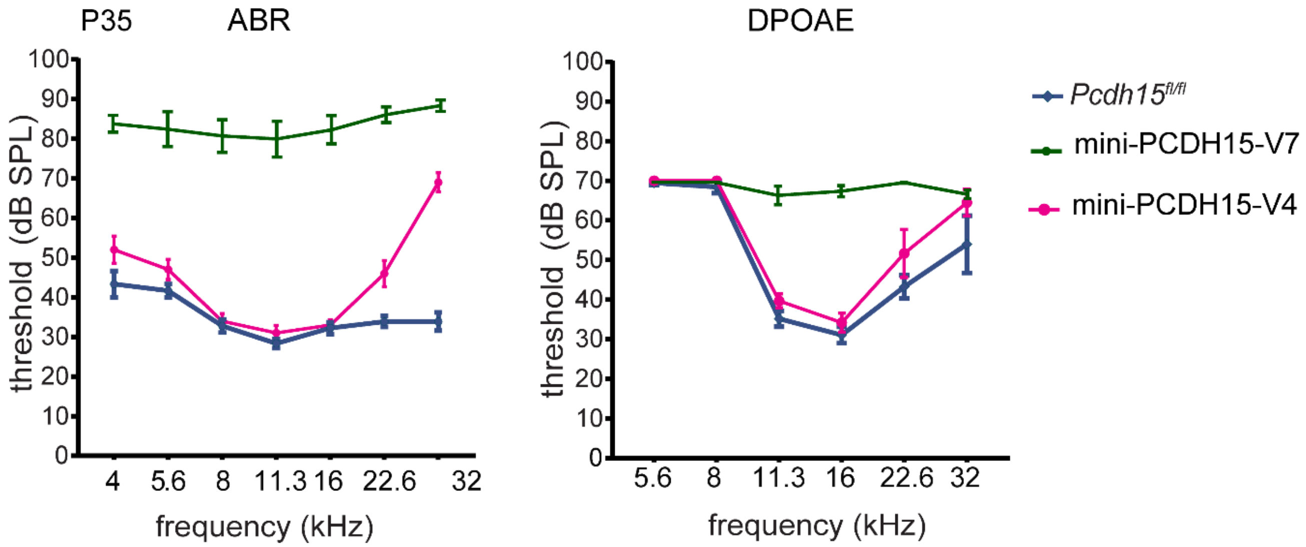

Supplementary Fig. 9.

Auditory brainstem response (ABR) and distortion-product otoacoustic emission (DPOAE) in *Pcdh15*$^{fl/fl}$ hearing control mice injected with AAV-mini-PCDH15-V4 (pink; n=5) or -V7 (green; n=13). Control mice injected with AAV-mini-PCDH15-V7 exhibited elevated ABR thresholds, suggesting toxicity. Mice injected with AAV-mini-PCDH15-V4 had normal hearing at most frequencies, indicating little or no toxicity. Uninjected *Pcdh15*$^{fl/fl}$ control mice thresholds indicated as blue (n=9). Data are presented as mean ± SEM. Source data are provided as a Source Data file.

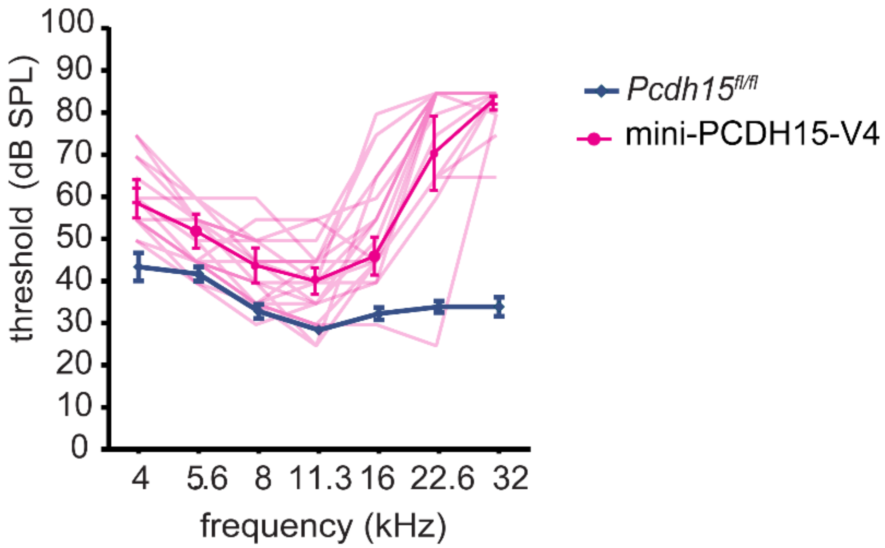

Supplementary Fig. 10.

Auditory brainstem response (ABR) threshold as a function of frequency for P35 uninjected *Pcdh15*$^{fl/fl}$ hearing control mice (blue; n=9) or *Pcdh15*$^{fl/fl}$*,Myo15-Cre*$^{+/-}$ mice injected with AAV-mini-PCDH15-V4 and AAV-mini-HA-PCDH15-V4 (pink; n=21). Light traces show individual recordings for treated mice. Data are presented as mean ± SEM. Source data are provided as a Source Data file.

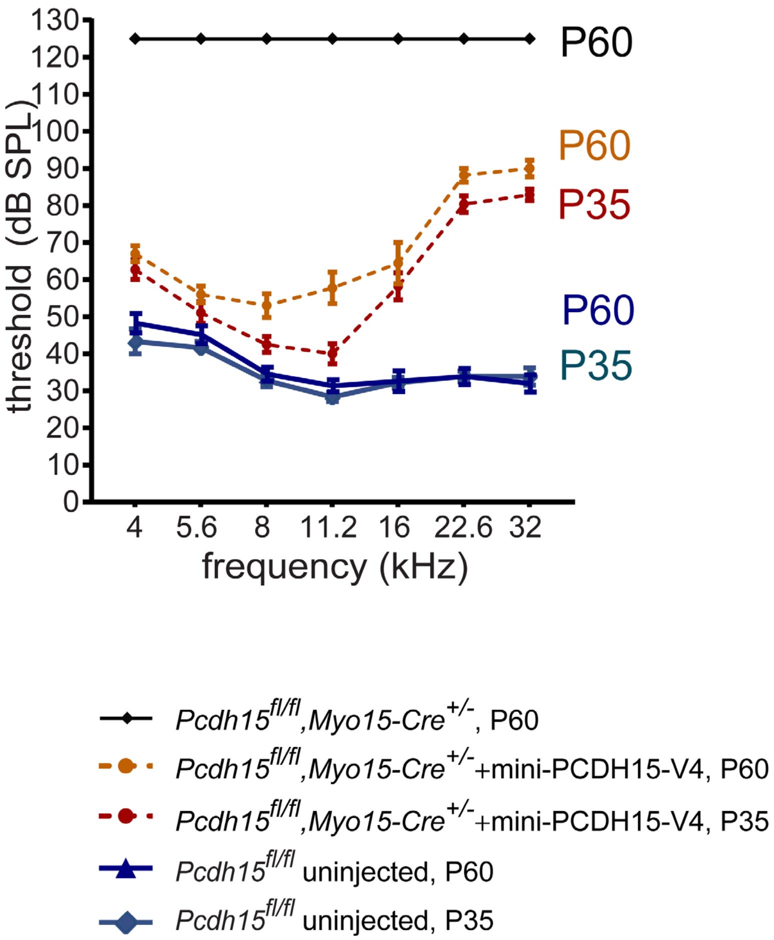

Supplementary Fig. 11.

Rescue of hearing with mini-PCDH15-V4 is mostly preserved at P60. Average auditory brainstem response (ABR) thresholds of mini-PCDH15-V4 treated *Myo15-Cre*$^{+}$ conditional knockout mice at P35 (n=14) (red dashed) and P60 (n=10) (yellow dashed), compared to normal thresholds of control P35 (n=9) (blue solid) and P60 (n=8) (dark blue solid) untreated *Pcdh15*$^{fl/fl}$ mice, and to untreated deaf P60 (black solid) *Pcdh15*$^{fl/fl}$,*Myo15-Cre*$^{+/-}$ cKO mice (n=5). Data are presented as mean ± SEM. Source data are provided as a Source Data file.

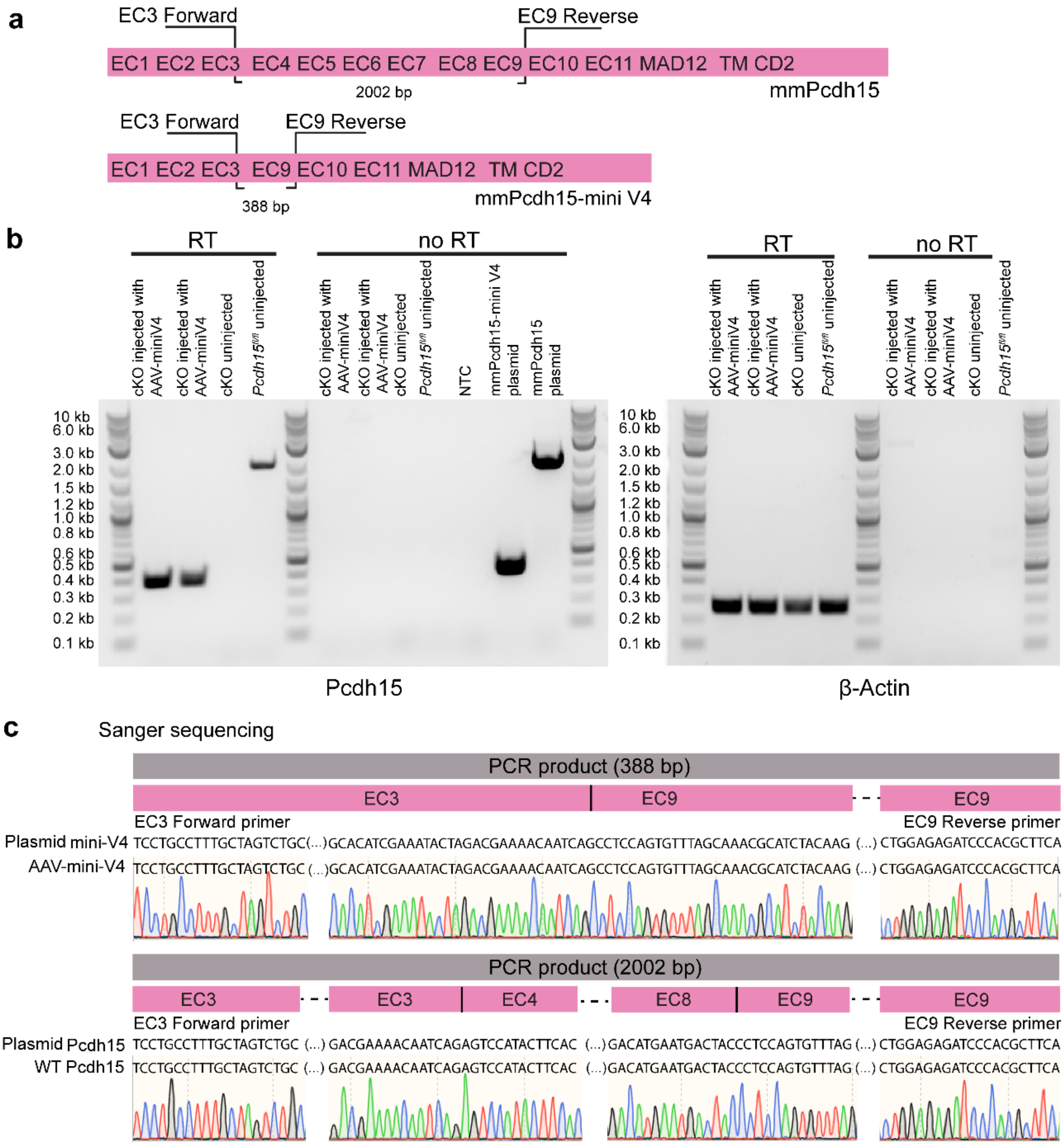

Supplementary Fig. 12.

RT-PCR showing the presence of *mini-PCDH15-V4* transcript in treated mice. a Schematic demonstrating the binding sites of primers used in cDNA amplification for wild-type (upper) versus *mini-PCDH15-V4* (lower) cDNA. Expected amplicon sizes (2002 versus 388 bp) are indicated. **b** RT-PCR of *Pcdh15* transcript from control *Pcdh15*$^{fl/fl}$ mice and from uninjected or injected *Myo15-Cre* conditional knockout mice, run on a 1.5% agarose gel stained with ethidium bromide. Short transcripts were detected in mini-PCDH15-V4 injected mice. Samples not treated with reverse transcriptase demonstrate no amplification. RT-PCR of beta-actin (right) shows similar amplification levels among samples. **c** Sanger sequencing confirmed the correct EC3-EC9 junction sequence in the cDNA, which was absent in the wild-type (WT Pcdh15) cDNA. Source data are provided as a Source Data file.

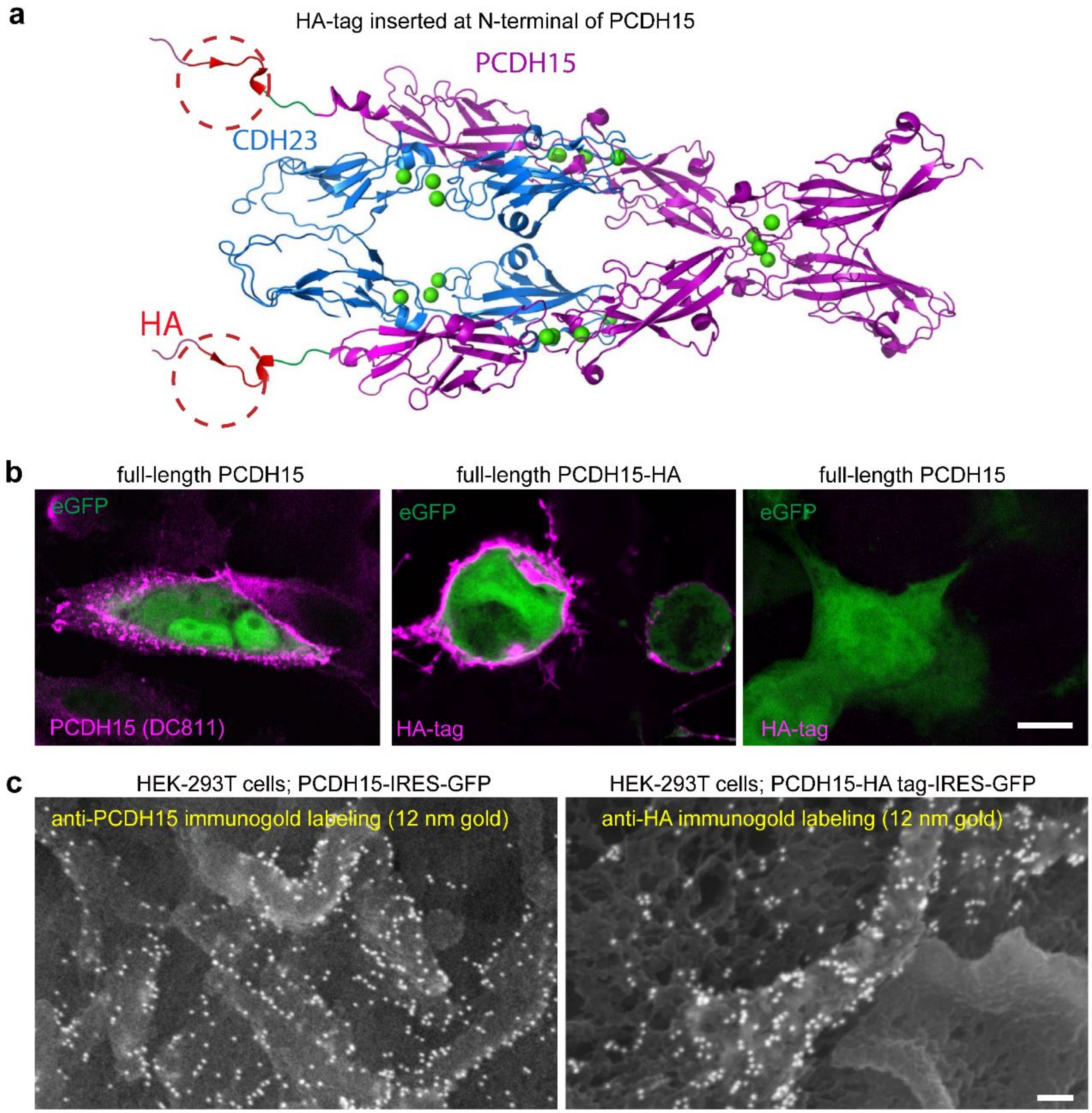

Supplementary Fig. 13.

Fluorescence and immunogold labeling show similar trafficking of HA-tagged PCDH15 and wild-type PCDH15 in HEK293T cells. a X-ray structure of PCDH15 bound to CDH23 with the predicted structure of the HA peptide (red circle) added to the N-terminus of PCDH15. The PCDH15 dimer (EC1-3; magenta) binds to the CDH23 dimer (EC1-2; blue) in a dual handshake configuration (adapted from Choudhary et al., 2020). **b** PCDH15-IRES-eGFP plasmids expressing the CD2 isoform of full-length PCDH15 (with or without an N-terminal HA tag) were transfected into HEK293T cells. Immunostaining was performed with anti-PCDH15 (left panel) or anti-HA (right and middle panels) antibodies. Representative confocal images demonstrate normal PCDH15 trafficking to the cell membrane either with or without the N-terminal HA tag. PCDH15 label was only observed on PCDH15-transfected cells (eGFP, green). **c** Representative immunogold scanning electron micrographs show extracellular labeling of PCDH15 with anti-PCDH15 (left) and anti-HA (right) antibodies, indicating normal protein transport to the cell membrane and no obvious disruption due to the HA tag. Scale bars: **b** 10 μm, **c** 100 nm.

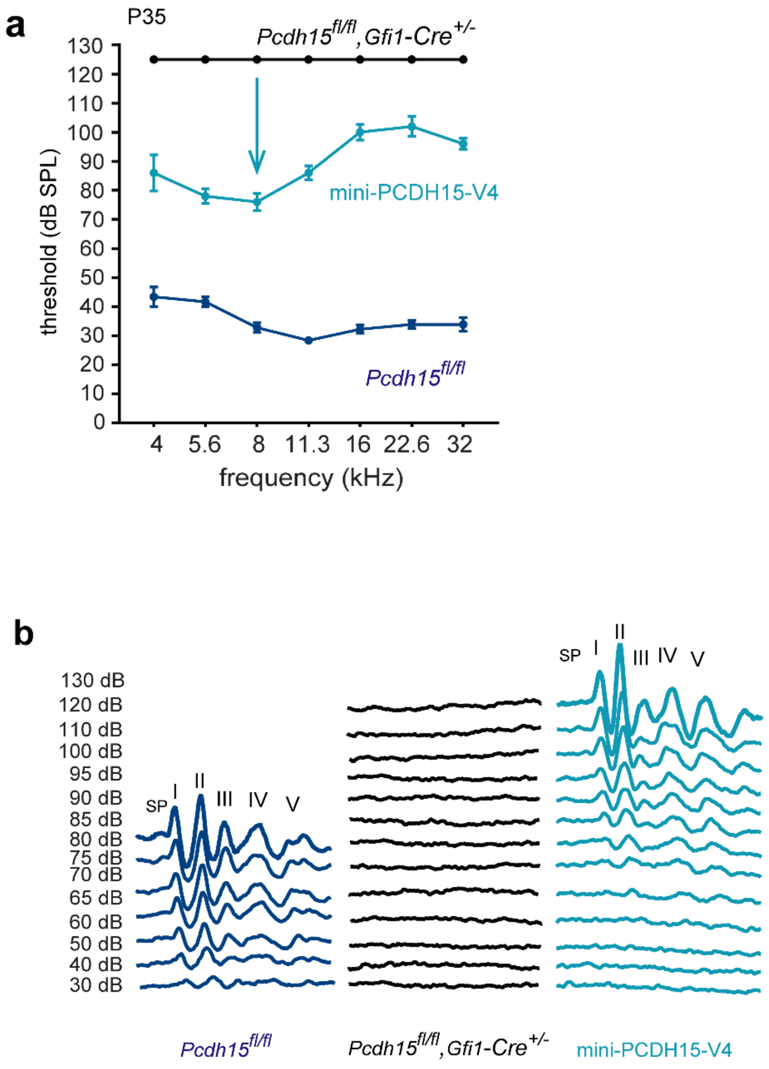

Supplementary Fig. 14.

AAV-mini-PCDH15-V4s rescues hearing in the *Gfi1-Cre* mouse model of USH1F. **a** Average auditory brainstem response (ABR) thresholds as a function of frequency in P35 mice. *Pcdh15*$^{fl/fl}$,*Gfi1-Cre*$^{+/-}$ deaf control mice (black; n=6) showed a profound hearing loss at P35 relative to *Pcdh15*$^{fl/fl}$ hearing control mice (dark blue; n=9). *Pcdh15*$^{fl/fl}$, *Gfi1-Cre*$^{+/-}$ conditional knockout mice treated with AAV-mini-PCDH15-V4 showed ~50 dB improvement of ABR thresholds (light blue; n=5). Data are presented as mean ± SEM. **b** Representative ABR traces from P35 hearing control mice lacking Cre, from uninjected knockout mice and from knockout mice injected with AAV-mini-PCDH15-V4. SP, Summating potential. Source data are provided as a Source Data file

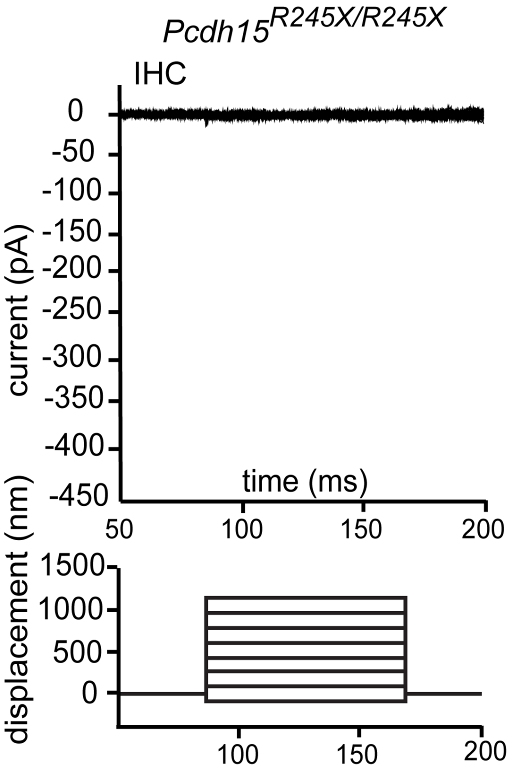

Supplementary Fig. 15.

Lack of mechanotransduction in Pcdh15^{R245X} homozygous knockout mice. No current was detected in *Pcdh15$^{R245X/R245X}$* (n=3) mice measured at P10-P17 (top) in response to bundle deflections of −175 to +1135-nm (bottom). IHC, inner hair cell (

Supplementary Table 1.

Protein sequence information of the mouse mini-PCDH15 variants. The middle region of eight mouse mini-PCDH15s exhibited varying compositions, but all mini-PCDH15s retained the EC1-3 repeats and the EC11-MAD12-CD2 without any changes.

Version	Protein sequence
mm.mini-PCDH15-V1	QYDDDWQYEDCKLARGGPPATIVAIDEESRNGTILVDNMLIKGTAGGPDPTIELSLKDNVDYWVLLDPVKQMLFLNSTGRVLDR DPPMNIHSIVVQVQCVNKKVGTVIYHEVRIVVRDRNDNSPTFKHESYYATVNELTPVGTTIFTGFSGDNGATDIDDGPNGQIEYVI QYNPEDPTSNDTFEIPLMLTGNVVLRKRLNYEDKTRYYVIIQANDRAQNLNERRTTTTTLTVDVLDGDDLGPMFLPCVLVPNTRD CRPLTYQAAIPELRTPEELNPILVTPPIQAIDQDRNIQPPSDRPGILYSILVGTPEDYPRFFHMHPRTAELTLLEPVNRDFHQKFDLVI KAEQDNGHPLPAFASLHIEILDENNQTPTFPEISYDVYVYTDMSPGDSVIQLTAVDADEGSNGEISYEILVGGKGDFVINKTTGLVSI APGVELIVGQTYALTVQASDNAPPAERRHSICTVYIEVLPPNNQSPPRFPQLMYSLEVSEAMRIGAILLNLQATDREGDPITYAIEN GDPQRVFNLSETTGILSLGKALDRESTDRYILIVTASDGRPDGTSTATVNIVVTDVNDNPPVFSKRIYKGMVAPDAVKGTPITTVYA EDADPPGMPASRVRYRVDDVQFPYPASIFDVEEDSGRVVTRVNLNEEPTTIFKLVVVAFDDGEPVMSSSATVRILVLHPGEIPRFTQ EEYRPPPVSELAARGTVVGVISAAAINQSIVYSIVAGNEEDKFGINNVTGVIYVNSPLDYETRTSYVLRVQADSLEVVLANLRVPSKS NTAKVYIEIQDENDHPPVFQKKFYIGGVSEDARMFASVLRVKATDRDTGNYSAMAYRLIIPPIKEGKEGFVVETYTGLIKTAMLFH NMRRSYFKFQVIATDDYGKGLSGKADVLVSVVNQLDMQVIVSNVPPTLVEKKIEDLTEILDRYVQEQIPGAKVVVESIGARRHGD AYSLEDYSKCDLTVYAIDPQTNRAIDRNELFKFLDGKLLDINKDFQPYYGEGGRILEIRTPEAVTSIKKRGESLGYTEGALLALAFIIIL CCIPAILVVLVSYRQFKVRQAECTKTARIQSAMPAAKPAAPVPAAPAPPPPPPPPPPGAHLYEELGESAMHKYEMPQYGSRRRLLP PAGQEEYGEVIGEAEEEYEEEEVEPEKVKKPKVEIREPSEEEVVVTVEKPPAAEPTYPTWKRARIFPMIFKKVRGLAEKRGIDLEGEE WRRRLDEEDKDYLQLTLDQEEATESTVESEEESSDYTEYTETESEFSESETTEESESETPSEEAEESSTPESEESESTESEGEKARKNIVL ARRRPVVEEIQEVKGKREEPPVEEEEEPPLEEEERAEEGEESEAAPMDESTDLEAQDVPEEGSAESVSMERGVESEESESELSSSSSTSE SLSGGPWGFQVPEYDRRKDEEPKKSPGANSEGYNTAL
mm.mini-PCDH15-V2	QYDDDWQYEDCKLARGGPPATIVAIDEESRNGTILVDNMLIKGTAGGPDPTIELSLKDNVDYWVLLDPVKQMLFLNSTGRVLDR DPPMNIHSIVVQVQCVNKKVGTVIYHEVRIVVRDRNDNSPTFKHESYYATVNELTPVGTTIFTGFSGDNGATDIDDGPNGQIEYVI QYNPEDPTSNDTFEIPLMLTGNVVLRKRLNYEDKTRYYVIIQANDRAQNLNERRTTTTTLTVDVLDGDDLGPMFLPCVLVPNTRD CRPLTYQAAIPELRTPEELNPILVTPPIQAIDQDRNIQPPSDRPGILYSILVGTPEDYPRFFHMHPRTAELTLLEPVNRDFHQKFDLVI KAEQDNGHPLPAFASLHIEILDENNQSPYFTMPSYQGYILESAPVGATISESLNLTTPLRIVALDKDIEDTKDPELHLFLNDYTSVFT VTPTGITRYLTLLQPVDREEQQTYTFLITAFDGVQESEPVVVNIRVMDANDNSPVFTNSTYTVVVEENLPAGTSFLQIEAKDVDLGA NVSYRIRSPEVKHLFALHPFTGELSLLRSLDYEAFPDQEASITFLVEAFDIYGTMPPGIATVTVIVKDMNDYPPVFSKRIYKGMVAPD AVKGTPITTVYAEDADPPGMPASRVRYRVDDVQFPYPASIFDVEEDSGRVVTRVNLNEEPTTIFKLVVVAFDDGEPVMSSSATVRIL VLHPGEIPRFTQEEYRPPPVSELAARGTVVGVISAAAINQSIVYSIVAGNEEDKFGINNVTGVIYVNSPLDYETRTSYVLRVQADSLE VVLANLRVPSKSNTAKVYIEIQDENDHPPVFQKKFYIGGVSEDARMFASVLRVKATDRDTGNYSAMAYRLIIPPIKEGKEGFVVET YTGLIKTAMLFHNMRRSYFKFQVIATDDYGKGLSGKADVLVSVVNQLDMQVIVSNVPPTLVEKKIEDLTEILDRYVQEQIPGAKV VVESIGARRHGDAYSLEDYSKCDLTVYAIDPQTNRAIDRNELFKFLDGKLLDINKDFQPYYGEGGRILEIRTPEAVTSIKKRGESLGY TEGALLALAFIIILCCIPAILVVLVSYRQFKVRQAECTKTARIQSAMPAAKPAAPVPAAPAPPPPPPPPPPGAHLYEELGESAMHKY EMPQYGSRRRLLPPAGQEEYGEVIGEAEEEYEEEEVEPEKVKKPKVEIREPSEEEVVVTVEKPPAAEPTYPTWKRARIFPMIFKKVRG LAEKRGIDLEGEEWRRRLDEEDKDYLQLTLDQEEATESTVESEEESSDYTEYTETESEFSESETTEESESETPSEEAEESSTPESEESESTE SEGEKARKNIVLARRRPVVEEIQEVKGKREEPPVEEEEEPPLEEEERAEEGEESEAAPMDESTDLEAQDVPEEGSAESVSMERGVESE ESESELSSSSSTSESLSGGPWGFQVPEYDRRKDEEPKKSPGANSEGYNTAL
mm.mini-PCDH15-V3	QYDDDWQYEDCKLARGGPPATIVAIDEESRNGTILVDNMLIKGTAGGPDPTIELSLKDNVDYWVLLDPVKQMLFLNSTGRVLDR DPPMNIHSIVVQVQCVNKKVGTVIYHEVRIVVRDRNDNSPTFKHESYYATVNELTPVGTTIFTGFSGDNGATDIDDGPNGQIEYVI QYNPEDPTSNDTFEIPLMLTGNVVLRKRLNYEDKTRYYVIIQANDRAQNLNERRTTTTTLTVDVLDGDDLGPMFLPCVLVPNTRD CRPLTYQAAIPELRTPEELNPILVTPPIQAIDQDRNIQPPSDRPGILYSILVGTPEDYPRFFHMHPRTAELTLLEPVNRDFHQKFDLVI KAEQDNGHPLPAFASLHIEILDENNQSPVFTNSTYTVVVEENLPAGTSFLQIEAKDVDLGANVSYRIRSPEVKHLFALHPFTGELSL LRSLDYEAFPDQEASITFLVEAFDIYGTMPPGIATVTVIVKDMNDYPPVFSKRIYKGMVAPDAVKGTPITTVYAEDADPPGMPASR VRYRVDDVQFPYPASIFDVEEDSGRVVTRVNLNEEPTTIFKLVVVAFDDGEPVMSSSATVRILVLHPGEIPRFTQEEYRPPPVSELAA RGTVVGVISAAAINQSIVYSIVAGNEEDKFGINNVTGVIYVNSPLDYETRTSYVLRVQADSLEVVLANLRVPSKSNTAKVYIEIQDE NDHPPVFQKKFYIGGVSEDARMFASVLRVKATDRDTGNYSAMAYRLIIPPIKEGKEGFVVETYTGLIKTAMLFHNMRRSYFKFQVI ATDDYGKGLSGKADVLVSVVNQLDMQVIVSNVPPTLVEKKIEDLTEILDRYVQEQIPGAKVVVESIGARRHGDAYSLEDYSKCDL TVYAIDPQTNRAIDRNELFKFLDGKLLDINKDFQPYYGEGGRILEIRTPEAVTSIKKRGESLGYTEGALLALAFIIILCCIPAILVVLVS YRQFKVRQAECTKTARIQSAMPAAKPAAPVPAAPAPPPPPPPPPPGAHLYEELGESAMHKYEMPQYGSRRRLLPPAGQEEYGEVI GEAEEEYEEEEVEPEKVKKPKVEIREPSEEEVVVTVEKPPAAEPTYPTWKRARIFPMIFKKVRGLAEKRGIDLEGEEWRRRLDEEDK DYLQLTLDQEEATESTVESEEESSDYTEYTETESEFSESETTEESESETPSEEAEESSTPESEESESTESEGEKARKNIVLARRRPVVEEIQE VKGKREEPPVEEEEEPPLEEEERAEEGEESEAAPMDESTDLEAQDVPEEGSAESVSMERGVESEESESELSSSSSTSESLSGGPWGFQV PEYDRRKDEEPKKSPGANSEGYNTAL

mm.mini-PCDH15-V4	QYDDDWQYEDCKLARGGPPATIVAIDEESRNGTILVDNMLIKGTAGGPDPTIELSLKDNVDYWVLLDPVKQMLFLNSTGRVLDR<br>DPPMNIHSIVVQVQCVNKKVGTVIYHEVRIVVRDRNDNSPTFKHESYYATVNELTPVGTTIFTGFSGDNGATDIDDGPNGQIEYVI<br>QYNPEDPTSNDTFEIPLMLTGNVVLRKRLNYEDKTRYYVIIQANDRAQNLNERRTTTTTLTVDVLDGDDLGPMFLPCVLVPNTRD<br>CRPLTYQAAIPELRTPEELNPILVTPPIQAIDQDRNIQPPSDRPGILYSILVGTPEDYPRFFHMHPRTAELTLLEPVNRDFHQKFDLVI<br>KAEQDNGHPLPAFASLHIEILDENNQPPVFSKRIYKGMVAPDAVKGTPITTVYAEDADPPGMPASRVRYRVDDVQFPYPASIFDV<br>EEDSGRVVTRVNLNEEPTTIFKLVVVAFDDGEPVMSSSATVRILVLHPGEIPRFTQEEYRPPPVSELAARGTVVGVISAAAINQSIVYS<br>IVAGNEEDKFGINNVTGVIYVNSPLDYETRTSYVLRVQADSLEVVLANLRVPSKSNTAKVYIEIQDENDHPPVFQKKFYIGGVSED<br>ARMFASVLRVKATDRDTGNYSAMAYRLIIPPIKEGKEGFVVETYTGLIKTAMLFHNMRRSYFKFQVIATDDYGKGLSGKADVLVS<br>VVNQLDMQVIVSNVPPTLVEKKIEDLTEILDRYVQEQIPGAKVVVESIGARRHGDAYSLEDYSKCDLTVYAIDPQTNRAIDRNELF<br>KFLDGKLLDINKDFQPYYGEGGRILEIRTPEAVTSIKKRGESLGYTEGALLALAFIIILCCIPAILVVLVSYRQFKVRQAECTKTARIQS<br>AMPAAKPAAPVPAAPAPPPPPPPPPPGAHLYEELGESAMHKYEMPQYGSRRRLLPPAGQEEYGEVIGEAEEEYEEEEVEPEKVKK<br>PKVEIREPSEEEVVVTVEKPPAAEPTYPTWKRARIFPMIFKKVRGLAEKRGIDLEGEEWRRRLDEEDKDYLQLTLDQEEATESTVESE<br>EESSDYTEYTETESEFSESETTEESESETPSEEAEESSTPESEESESTESEGEKARKNIVLARRRPVVEEIQEVKGKREEPPVEEEEEPPLEE<br>EERAEEGEESEAAPMDESTDLEAQDVPEEGSAESVSMERGVESEESESELSSSSSTSESLSGGPWGFQVPEYDRRKDEEPKKSPGANS<br>EGYNTAL
mm.mini-PCDH15-V5	QYDDDWQYEDCKLARGGPPATIVAIDEESRNGTILVDNMLIKGTAGGPDPTIELSLKDNVDYWVLLDPVKQMLFLNSTGRVLDR<br>DPPMNIHSIVVQVQCVNKKVGTVIYHEVRIVVRDRNDNSPTFKHESYYATVNELTPVGTTIFTGFSGDNGATDIDDGPNGQIEYVI<br>QYNPEDPTSNDTFEIPLMLTGNVVLRKRLNYEDKTRYYVIIQANDRAQNLNERRTTTTTLTVDVLDGDDLGPMFLPCVLVPNTRD<br>CRPLTYQAAIPELRTPEELNPILVTPPIQAIDQDRNIQPPSDRPGILYSILVGTPEDYPRFFHMHPRTAELTLLEPVNRDFHQKFDLVI<br>KAEQDNGHPLPAFASLHIEILDENNQTPTFPEISYDVYVYTDMSPGDSVIQLTAVDADEGSNGEISYEILVGGKGDFVINKTTGLVSI<br>APGVELIVGQTYALTVQASDNAPPAERRHSICTVYIEVLPPNNQSPPRFPQLMYSLEVSEAMRIGAILLNLQATDREGDPITYAIEN<br>GDPQRVFNLSETTGILSLGKALDRESTDRYILIVTASDGRPDGTSTATVNIVVTDVNDNAPVFDPYLPRNLSVVEEEANAFVGQVR<br>ATDPDAGINGQVHYSLGNFNNLFRITSNGSIYTAVKLNREARDHYELVVVATDGAVHPRHSTLTLYIKVLDIDDNPPVFQKKFYIG<br>GVSEDARMFASVLRVKATDRDTGNYSAMAYRLIIPPIKEGKEGFVVETYTGLIKTAMLFHNMRRSYFKFQVIATDDYGKGLSGKA<br>DVLVSVVNQLDMQVIVSNVPPTLVEKKIEDLTEILDRYVQEQIPGAKVVVESIGARRHGDAYSLEDYSKCDLTVYAIDPQTNRAID<br>RNELFKFLDGKLLDINKDFQPYYGEGGRILEIRTPEAVTSIKKRGESLGYTEGALLALAFIIILCCIPAILVVLVSYRQFKVRQAECTKT<br>ARIQSAMPAAKPAAPVPAAPAPPPPPPPPPPGAHLYEELGESAMHKYEMPQYGSRRRLLPPAGQEEYGEVIGEAEEEYEEEEVEPE<br>KVKKPKVEIREPSEEEVVVTVEKPPAAEPTYPTWKRARIFPMIFKKVRGLAEKRGIDLEGEEWRRRLDEEDKDYLQLTLDQEEATES<br>TVESEEESSDYTEYTETESEFSESETTEESESETPSEEAEESSTPESEESESTESEGEKARKNIVLARRRPVVEEIQEVKGKREEPPVEEEEE<br>PPLEEEERAEEGEESEAAPMDESTDLEAQDVPEEGSAESVSMERGVESEESESELSSSSSTSESLSGGPWGFQVPEYDRRKDEEPKKSP<br>GANSEGYNTAL
mm.mini-PCDH15-V6	QYDDDWQYEDCKLARGGPPATIVAIDEESRNGTILVDNMLIKGTAGGPDPTIELSLKDNVDYWVLLDPVKQMLFLNSTGRVLDR<br>DPPMNIHSIVVQVQCVNKKVGTVIYHEVRIVVRDRNDNSPTFKHESYYATVNELTPVGTTIFTGFSGDNGATDIDDGPNGQIEYVI<br>QYNPEDPTSNDTFEIPLMLTGNVVLRKRLNYEDKTRYYVIIQANDRAQNLNERRTTTTTLTVDVLDGDDLGPMFLPCVLVPNTRD<br>CRPLTYQAAIPELRTPEELNPILVTPPIQAIDQDRNIQPPSDRPGILYSILVGTPEDYPRFFHMHPRTAELTLLEPVNRDFHQKFDLVI<br>KAEQDNGHPLPAFASLHIEILDENNQSPYFTMPSYQGYILESAPVGATISESLNLTTPLRIVALDKDIEDTKDPELHLFLNDYTSVFT<br>VTPTGITRYLTLLQPVDREEQQTYTFLITAFDGVQESEPVVVNIRVMDANDNAPVFDPYLPRNLSVVEEEANAFVGQVRATDPDA<br>GINGQVHYSLGNFNNLFRITSNGSIYTAVKLNREARDHYELVVVATDGAVHPRHSTLTLYIKVLDIDDNSPVFTNSTYTVVVEENL<br>PAGTSFLQIEAKDVDLGANVSYRIRSPEVKHLFALHPFTGELSLLRSLDYEAFPDQEASITFLVEAFDIYGTMPPGIATVTVIVKDMN<br>DYPPVFQKKFYIGGVSEDARMFASVLRVKATDRDTGNYSAMAYRLIIPPIKEGKEGFVVETYTGLIKTAMLFHNMRRSYFKFQVIA<br>TDDYGKGLSGKADVLVSVVNQLDMQVIVSNVPPTLVEKKIEDLTEILDRYVQEQIPGAKVVVESIGARRHGDAYSLEDYSKCDLT<br>VYAIDPQTNRAIDRNELFKFLDGKLLDINKDFQPYYGEGGRILEIRTPEAVTSIKKRGESLGYTEGALLALAFIIILCCIPAILVVLVSY<br>RQFKVRQAECTKTARIQSAMPAAKPAAPVPAAPAPPPPPPPPPPGAHLYEELGESAMHKYEMPQYGSRRRLLPPAGQEEYGEVIG<br>EAEEEYEEEEVEPEKVKKPKVEIREPSEEEVVVTVEKPPAAEPTYPTWKRARIFPMIFKKVRGLAEKRGIDLEGEEWRRRLDEEDKD<br>YLQLTLDQEEATESTVESEEESSDYTEYTETESEFSESETTEESESETPSEEAEESSTPESEESESTESEGEKARKNIVLARRRPVVEEIQEV<br>KGKREEPPVEEEEEPPLEEEERAEEGEESEAAPMDESTDLEAQDVPEEGSAESVSMERGVESEESESELSSSSSTSESLSGGPWGFQVP<br>EYDRRKDEEPKKSPGANSEGYNTAL

mm.mini-PCDH15-V7	QYDDDWQYEDCKLARGGPPATIVAIDEESRNGTILVDNMLIKGTAGGPDPTIELSLKDNVDYWVLLDPVKQMLFLNSTGRVLDR<br>DPPMNIHSIVVQVQCVNKKVGTVIYHEVRIVVRDRNDNSPTFKHESYYATVNELTPVGTTIFTGFSGDNGATDIDDGPNGQIEYVI<br>QYNPEDPTSNDTFEIPLMLTGNVVLRKRLNYEDKTRYYVIIQANDRAQNLNERRTTTTTLTVDVLDGDDLGPMFLPCVLVPNTRD<br>CRPLTYQAAIPELRTPEELNPILVTPPIQAIDQDRNIQPPSDRPGILYSILVGTPEDYPRFFHMHPRTAELTLLEPVNRDFHQKFDLVI<br>KAEQDNGHPLPAFASLHIEILDENNQAPVFDPYLPRNLSVVEEEANAFVGQVRATDPDAGINGQVHYSLGNFNNLFRITSNGSIY<br>TAVKLNREARDHYELVVVATDGAVHPRHSTLTLYIKVLDIDDNSPVFTNSTYTVVVEENLPAGTSFLQIEAKDVDLGANVSYRIRS<br>PEVKHLFALHPFTGELSLLRSLDYEAFPDQEASITFLVEAFDIYGTMPPGIATVTVIVKDMNDYPPVFQKKFYIGGVSEDARMFASV<br>LRVKATDRDTGNYSAMAYRLIIPPIKEGKEGFVVETYTGLIKTAMLFHNMRRSYFKFQVIATDDYGKGLSGKADVLVSVVNQLDM<br>QVIVSNVPPTLVEKKIEDLTEILDRYVQEQIPGAKVVVESIGARRHGDAYSLEDYSKCDLTVYAIDPQTNRAIDRNELFKFLDGKLL<br>DINKDFQPYYGEGGRILEIRTPEAVTSIKKRGESLGYTEGALLALAFIIILCCIPAILVVLVSYRQFKVRQAECTKTARIQSAMPAAKP<br>AAPVPAAPAPPPPPPPPPPGAHLYEELGESAMHKYEMPQYGSRRRLLPPAGQEEYGEVIGEAEEEYEEEEVEPEKVKKPKVEIREPS<br>EEEVVVVTVEKPPAAEPTYPTWKRARIFPMIFKKVRGLAEKRGIDLEGEEWRRRLDEEDKDYLQLTLDQEEATESTVESEEESSDYTE<br>YTETESEFSESETTEESESETPSEEAEESSTPESEESESTESEGEKARKNIVLARRRPVVEEIQEVKGKREEPPVEEEEEPPLEEEERAEEG<br>EESEAAPMDESTDLEAQDVPEEGSAESVSMERGVESEESESELSSSSSTSESLSGGPWGFQVPEYDRRKDEEPKKSPGANSEGYNTAL
mm.mini-PCDH15-V8	QYDDDWQYEDCKLARGGPPATIVAIDEESRNGTILVDNMLIKGTAGGPDPTIELSLKDNVDYWVLLDPVKQMLFLNSTGRVLDR<br>DPPMNIHSIVVQVQCVNKKVGTVIYHEVRIVVRDRNDNSPTFKHESYYATVNELTPVGTTIFTGFSGDNGATDIDDGPNGQIEYVI<br>QYNPEDPTSNDTFEIPLMLTGNVVLRKRLNYEDKTRYYVIIQANDRAQNLNERRTTTTTLTVDVLDGDDLGPMFLPCVLVPNTRD<br>CRPLTYQAAIPELRTPEELNPILVTPPIQAIDQDRNIQPPSDRPGILYSILVGTPEDYPRFFHMHPRTAELTLLEPVNRDFHQKFDLVI<br>KAEQDNGHPLPAFASLHIEILDENNQSPYFTMPSYQGYILESAPVGATISESLNLTTPLRIVALDKDIEDTKDPELHLFLNDYTSVFT<br>VTPTGITRYLTLLQPVDREEQQTYTFLITAFDGVQESEPVVVNIRVMDANDNAPVFDPYLPRNLSVVEEEANAFVGQVRATDPDA<br>GINGQVHYSLGNFNNLFRITSNGSIYTAVKLNREARDHYELVVVATDGAVHPRHSTLTLYIKVLDIDDNPPVFQKKFYIGGVSEDA<br>RMFASVLRVKATDRDTGNYSAMAYRLIIPPIKEGKEGFVVETYTGLIKTAMLFHNMRRSYFKFQVIATDDYGKGLSGKADVLVSV<br>VNQLDMQVIVSNVPPTLVEKKIEDLTEILDRYVQEQIPGAKVVVESIGARRHGDAYSLEDYSKCDLTVYAIDPQTNRAIDRNELFKF<br>LDGKLLDINKDFQPYYGEGGRILEIRTPEAVTSIKKRGESLGYTEGALLALAFIIILCCIPAILVVLVSYRQFKVRQAECTKTARIQSAM<br>PAAKPAAPVPAAPAPPPPPPPPPPGAHLYEELGESAMHKYEMPQYGSRRRLLPPAGQEEYGEVIGEAEEEYEEEEVEPEKVKKPKV<br>EIREPSEEEVVVVTVEKPPAAEPTYPTWKRARIFPMIFKKVRGLAEKRGIDLEGEEWRRRLDEEDKDYLQLTLDQEEATESTVESEEES<br>SDYTEYTETESEFSESETTEESESETPSEEAEESSTPESEESESTESEGEKARKNIVLARRRPVVEEIQEVKGKREEPPVEEEEEPPLEEEER<br>AEEGEESEAAPMDESTDLEAQDVPEEGSAESVSMERGVESEESESELSSSSSTSESLSGGPWGFQVPEYDRRKDEEPKKSPGANSEGY<br>NTAL
