# Supplementary material for: Mini-PCDH15 gene therapy rescues hearing in a mouse model of Usher syndrome type 1F
Source: Nat Commun. 2023 Apr 26;14:2400. doi: 10.1038/s41467-023-38038-y (PMC10133396; doi:10.1038/s41467-023-38038-y)
Supplement: Supplementary file 3 — Reporting Summary [file 41467_2023_38038_MOESM3_ESM.pdf]

nature portfolio

Corresponding author(s): David P Corey

Last updated by author(s): Mar 15, 2023

Reporting Summary

Nature Portfolio wishes to improve the reproducibility of the work that we publish. This form provides structure for consistency and transparency in reporting. For further information on Nature Portfolio policies, see our Editorial Policies and the Editorial Policy Checklist.

Statistics

For all statistical analyses, confirm that the following items are present in the figure legend, table legend, main text, or Methods section.

n/a	Confirmed	
☐	☒	The exact sample size (*n*) for each experimental group/condition, given as a discrete number and unit of measurement
☐	☒	A statement on whether measurements were taken from distinct samples or whether the same sample was measured repeatedly
☐	☒	The statistical test(s) used AND whether they are one- or two-sided<br>*Only common tests should be described solely by name; describe more complex techniques in the Methods section.*
☐	☒	A description of all covariates tested
☐	☒	A description of any assumptions or corrections, such as tests of normality and adjustment for multiple comparisons
☐	☒	A full description of the statistical parameters including central tendency (e.g. means) or other basic estimates (e.g. regression coefficient) AND variation (e.g. standard deviation) or associated estimates of uncertainty (e.g. confidence intervals)
☐	☒	For null hypothesis testing, the test statistic (e.g. *F*, *t*, *r*) with confidence intervals, effect sizes, degrees of freedom and *P* value noted<br>*Give P values as exact values whenever suitable.*
☒	☐	For Bayesian analysis, information on the choice of priors and Markov chain Monte Carlo settings
☒	☐	For hierarchical and complex designs, identification of the appropriate level for tests and full reporting of outcomes
☒	☐	Estimates of effect sizes (e.g. Cohen's *d*, Pearson's *r*), indicating how they were calculated

Our web collection on statistics for biologists contains articles on many of the points above.

Software and code

Policy information about availability of computer code

Data collection	Confocal imaging data: Olympus FluoView 1000, Nikon Elements Acquisition Software AR 5.02, Leica Application Suite X (LAS X) 4.0.2, Unicorn 5.31
Data analysis	ABR Peak Analysis v1.1.1.9, Clampfit v 10.4.0.36 s, ColabFold v1.5.2: AlphaFold2, Chimera v1.17, PyMOL, Excel, GraphPad Prism v7.04, GraphPad Prism 9.5.0, Fiji 1.53, SnapGene 5.1, ASTRA 6.1, Adobe Illustrator 2022 v26.3.1., Excel 2016 v16.0.5378.1000

For manuscripts utilizing custom algorithms or software that are central to the research but not yet described in published literature, software must be made available to editors and reviewers. We strongly encourage code deposition in a community repository (e.g. GitHub). See the Nature Portfolio guidelines for submitting code & software for further information.

Data

Policy information about availability of data

All manuscripts must include a data availability statement. This statement should provide the following information, where applicable:

- Accession codes, unique identifiers, or web links for publicly available datasets
- A description of any restrictions on data availability
- For clinical datasets or third party data, please ensure that the statement adheres to our policy

All data generated or analyzed during this study are included in this article and its supplementary information files. Source data are provided with this paper.

Human research participants

Policy information about studies involving human research participants and Sex and Gender in Research.

Reporting on sex and gender	No human subjects were used
Population characteristics	NA
Recruitment	NA
Ethics oversight	NA

Note that full information on the approval of the study protocol must also be provided in the manuscript.

Field-specific reporting

Please select the one below that is the best fit for your research. If you are not sure, read the appropriate sections before making your selection.

☒ Life sciences ☐ Behavioural & social sciences ☐ Ecological, evolutionary & environmental sciences

For a reference copy of the document with all sections, see nature.com/documents/nr-reporting-summary-flat.pdf

Life sciences study design

All studies must disclose on these points even when the disclosure is negative.

Sample size	Same size calculations were carried out using power analysis or based on similar studies published by other groups
Data exclusions	Only samples with significant dissection-related damage were removed from the analysis. No other data were excluded.
Replication	All experiments were successfully replicated, and represented results from at least three independent experiments on different days. For quantitative experiments a bigger sample size was selected where possible. We specify the number of biological replicates in the respective figure legends and in Statistics and reproducibility section
Randomization	Animals were randomly allocated to experimental groups in this study. Comparisons between the controls (untreated) and experimental (treated with AAV) was made based on genotype and whether animals were injected with AAV or not. Age was another experimental variable, it was controlled directly (i.e. controls and experimental groups were age-matched for experiments, or were litter mates when it was possible). Animals were raised under the same lighting, housing, and feeding conditions, and were on the same genetic background respectively . Both sexes of animals were used in each experiment and we did not observe any difference in the distribution of males/female in each genotype. Randomization was not a factor in the experiments, except for those involving animals in this study. The in vitro experiments were conducted on batches of transfected cells. Randomization is typically employed to counteract the influence of confounding variables that are unevenly distributed between comparison groups. However, in the context of the in vitro experiments carried out in this research, randomization was not applicable.
Blinding	Investigators were blinded only in experiments with ABR and DPOAE measurements, in which case they were blinded to the genotype of the animals. All other experiments were not blinded, given that subsequent experiments were carried out by the same researchers from the beginning and experimental conditions was required during data collection and evaluation.

Reporting for specific materials, systems and methods

We require information from authors about some types of materials, experimental systems and methods used in many studies. Here, indicate whether each material, system or method listed is relevant to your study. If you are not sure if a list item applies to your research, read the appropriate section before selecting a response.

Materials & experimental systems

n/a	Involved in the study	
☐	☒	Antibodies
☐	☒	Eukaryotic cell lines
☒	☐	Palaeontology and archaeology
☐	☒	Animals and other organisms
☒	☐	Clinical data
☒	☐	Dual use research of concern

Methods

n/a	Involved in the study	
☒	☐	ChIP-seq
☒	☐	Flow cytometry
☒	☐	MRI-based neuroimaging

Antibodies

Antibodies used	Rabbit Anti-HA tag antibody, Abcam, ab9110;<br>rabbit polyclonal anti-PCDH15 antibody DC811, custom-made;<br>12-nm Colloidal Gold AffiniPure Goat Anti-Rabbit IgG, Jackson ImmunoResearch, 111-205-144;<br>donkey anti-Rabbit IgG (H+L) Highly Cross-Adsorbed Secondary Antibody, Alexa Fluor™ 594, Invitrogen, A-21207
Validation	In the present study, the anti-PCDH15 custom-made antibody (DC811) was validated through immunofluorescence labeling, where a strong signal was detected at the tips of stereocilia in control cochleas, while no labeling was observed in Gfi1-Cre KO or R245X KO cochleas, thus confirming the antibody's specificity (Fig. 2f and Fig. 7c). The antibody was further validated through immunogold electron microscopy in KO and control mice (Fig. 2g), as well as in HEK cells transfected with PCDH15 plasmid (Supplementary figure 1). The specificity of the Rabbit anti-HA tag antibody ab9110 was validated by the manufacturer and in this study in vitro in HEK cells transfected with HA-tagged PCDH15 and untagged PCDH15 as a control. Immunolabeling with either anti-PCDH15 or anti-HA antibodies showed strong immunofluorescence labeling along the membranes, indicating that the HA tag co-localizes with PCDH15 (Supplementary Fig. 13b). These experiments were repeated using immunogold scanning electron microscopy labeling, which confirmed the specificity of the HA tag antibody (Supplementary Fig. 13c).

Eukaryotic cell lines

Policy information about cell lines and Sex and Gender in Research

Cell line source(s)	HEK293T cells from ATCC (cat #CRL-1573); HEK293NC cells provided by Dr. Joshua Sanes, Harvard University; Expi293F (Thermo Fisher, A14528), HeLa cells were ordered from ATCC (cat #CCL-2)
Authentication	Cells were authenticated by the supplier, no further authentication was done for cell lines.
Mycoplasma contamination	Beyond initial negative testing for mycoplasma contamination, HEK293T, HEK293NC, Expi293F cell lines were not tested for the remainder of the experiments. HeLa cells were tested for mycoplasma contamination every three months with a mycoplasma PCR detection kit (Sigma, MP0035-1KT).
Commonly misidentified lines<br>(See ICLAC register)	The study did not involve commonly misidentified lines.

Animals and other research organisms

Policy information about studies involving animals; ARRIVE guidelines recommended for reporting animal research, and Sex and Gender in Research

Laboratory animals	C57BL/6J; PCDH15fl/fl, mice on mixed C57BL/6J -129/Sv genetic backgrounds; Pcdh15fl/fl;Myo15-Cre mice on mixed C57BL/6J-129/Sv genetic backgrounds, Pcdh15fl/fl;Gfi1-Cre mice on mixed C57BL/6J -129/Sv genetic backgrounds, Pcdh15R245X/R245X on C57BL/6J genetic background. Age P0-P300. The mice were housed at the animal facility of the Harvard Medical School in a 12-hour-light/12-hour-dark cyclic environment. The temperature ranged from 73°F to 76°F, and the humidity from 30% to 40%.
Wild animals	The study did not involve wild animals.
Reporting on sex	The study did not take into account the sex of the mice used. Initial experiments indicated no sex-specific hearing phenotype, therefore male and female animals were utilized in the experiments described in the submission. No data was collected counting the amount of female vs male animals used for each experiment. Instead, only the total number of animals utilized was reported.
Field-collected samples	The study did not involve samples collected in the field.
Ethics oversight	All handling, maintenance, and experimental use of animals and protocols were approved by the Institutional Animal Care and Use Committee (protocol IS00001452) at Harvard Medical School, Boston, and were performed according to the NIH guidelines.

Note that full information on the approval of the study protocol must also be provided in the manuscript.
